# Process evaluation of the flucare cluster randomised controlled trial: assessing the implementation of a behaviour change intervention to increase influenza vaccination uptake among care home staff in England

**DOI:** 10.1186/s12913-025-13298-0

**Published:** 2025-08-21

**Authors:** Thando Katangwe-Chigamba, Faisal Alsaif, Adaku Anyiam-Osigwe, Veronica Bion, Allan Clark, Hilary Garrett, Alys Wyn Griffiths, Cecile Guillard, Amber Hammond, Richard Holland, Liz Jones, Amrish Patel, Jennifer Pitcher, Helen Risebro, Sion Scott, Carys Seeley, Erika Sims, Susan Stirling, Adam Wagner, David Wright, Linda Birt

**Affiliations:** 1 Norwich Clinical Trials Unit, University of East Anglia, Norwich, UK; 2 School of Pharmacy, University of East Anglia, Norwich, UK; 3 Norwich Medical School, University of East Anglia, Norwich, UK; 4 School of Medicine and Population Health, University of Sheffield, Sheffield, UK; 5 University of Exeter, Exeter Medical School, Exeter, UK; 6 School of Economics, University of East Anglia, Norwich, UK; 7 NIHR Applied Research Collaboration (ARC) East of England, Cambridge, UK; 8 School of Healthcare, University of Leicester, Leicester, UK

**Keywords:** Process evaluation, Residential homes, Nursing homes, Long-term care facilities, Staff, Employees, Community pharmacy

## Abstract

**Background:**

Influenza (flu) vaccination rates of Care home staff (CHS) in England are consistently lower (≈ 15% in 2023) than World Health Organisation recommendations (≥ 75%). The FluCare trial examined the effectiveness of a multi-component intervention (including on-site flu vaccination clinics, information materials including video, £850 incentive and monthly monitoring with feedback) designed to address known barriers to flu vaccine uptake amongst CHS. This paper reports an embedded process evaluation designed to understand implementation of the FluCare intervention and provide explanations for observed effects in the trial.

**Methods:**

The FluCare cluster randomised controlled trial was conducted between November 2022 and March 2023. A mixed methods process evaluation was conducted employing questionnaires, semi-structured interviews, video analytics (no. clicks and duration of view) and clinic logs (no. clinics delivered, days/time clinics were delivered, and no. staff vaccinated). CHS (including managers) and vaccination providers (pharmacists, nurses and general practitioners) were purposively and conveniently selected, respectively, for the interviews. Descriptive statistics were obtained for quantitative data, and qualitative data were analysed thematically.

**Results:**

FluCare intervention implementation varied across Care homes (CHs), with clinics and videos not being implemented in 35% and 43% of the intervention CHs respectively. In addition, clinic days and times varied depending on provider (pharmacy or general practice) and CH. Partial intervention implementation was partly influenced by managers’ engagement and sub-organisational cultures marked by negative narratives around vaccines. Contextual barriers included delivery of clinics late in the flu season. A greater indication of implementation fidelity was positively associated with change in staff attitudes and behaviours, with some getting vaccinated for the first time.

**Conclusions:**

Variation in implementation of the FluCare intervention provides an explanation for detecting a difference where the intervention was fully implemented in the main trial. Manager and leader engagement is vital for both successful implementation and staff engagement. Avoidable contextual barriers, such as late timing of clinics, must be addressed to enhance flu vaccination uptake by CHS. More work is needed to understand the role of CH leaders in influencing intervention implementation, sub-organisational cultures and vaccination attitudes.

**Trial registration:**

ISRCTN22729870. Registered on 24 August 2022.

**Supplementary Information:**

The online version contains supplementary material available at 10.1186/s12913-025-13298-0.

## Background

In the UK, seasonal influenza (flu) was estimated to cause 15,000 deaths in 2022–2023 [1]. Mortality rates for flu are highest in older people and those with co-morbidities, posing a major risk for care home (CH) residents [2, 3]. Vaccination is the primary form of protection against flu-related risks and complications e.g., pneumonia. In settings where multiple people live in proximity, such as residential and nursing CHs, these risks can be further mitigated by vaccinating care home staff (CHS) thus reducing cross-infection, particularly among vulnerable residents for whom vaccination is contraindicated or less effective [3–7]. Staff vaccination is associated with reductions in resident flu-like illness, hospitalisations and mortality [3–9]. In addition, improvements in staff health resulting from vaccination [10] decreases likelihood of absences and promotes continuity of care which itself is associated with increased resident health and quality of life [11].

The World Health Organization (WHO) recommends that at least 75% of health and social care staff are vaccinated against flu [12]. In England, this target is consistently met for National Health Service healthcare workers [13], but not for social care workers, including CHS. For example, in February 2023, only 15.7% of CHS were reported to have received the flu vaccination in England [14]. Therefore, there is an urgent need for an intervention to improve influenza vaccination uptake for CHS [15, 16]. Hesitancy to be vaccinated has been attributed to three main barriers: convenience, complacency and confidence [15]. Previous research has demonstrated provision of on-site clinics to be the most effective method for addressing barriers related to convenience and enhancing vaccine uptake [17, 18]. Other barriers such as not perceiving the need for vaccine [17, 19, 20] and lack of confidence in the vaccine, believing that vaccines are ineffective or can cause disease [17, 20–22], have been targeted by information provision, with effectiveness enhanced if information is tailored to identified barriers [23, 24]. However, research suggests that provision of on-site clinics, or information, in isolation is unlikely to increase staff vaccination uptake to meet the recommended WHO target of ≥ 75% [7, 18, 23, 25].

**Fig. 1:**
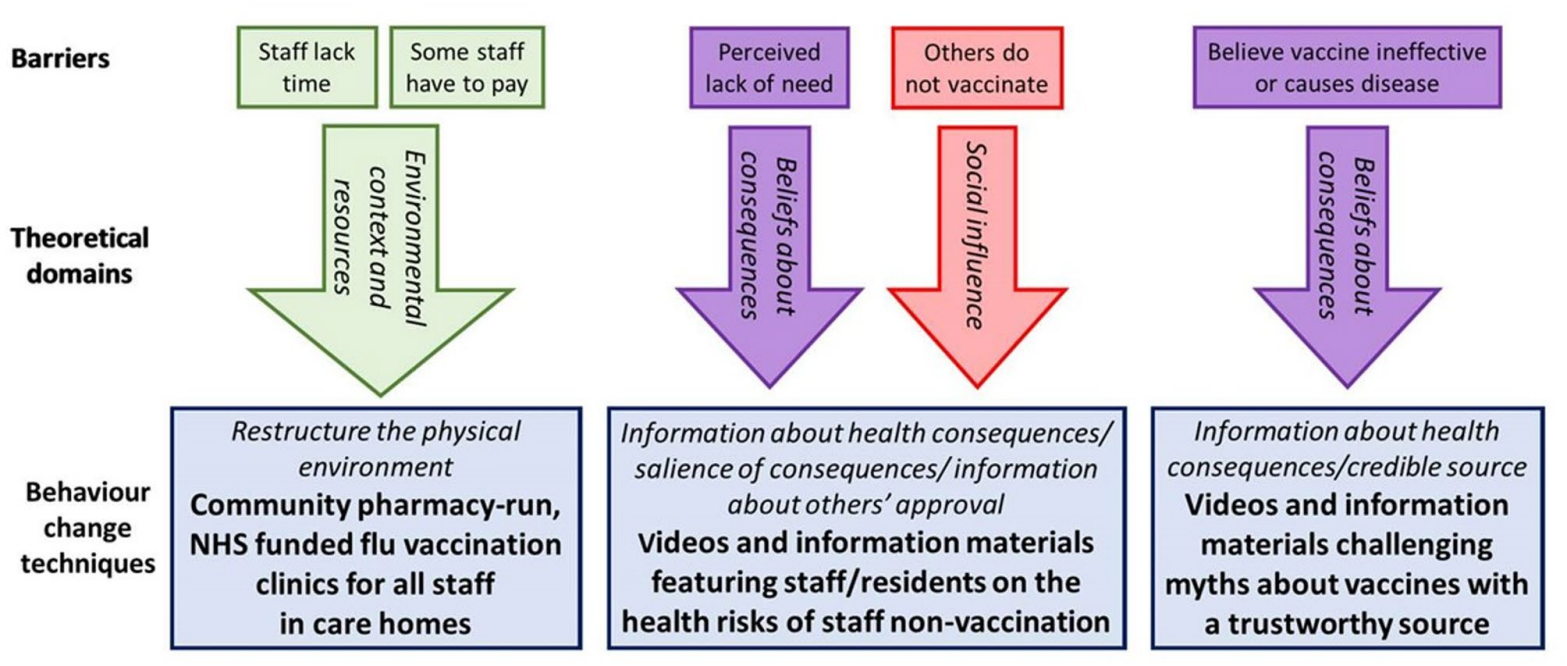
Relationship between behaviour change techniques, barriers, and theory

The National Institute for Health and Care Excellence recommends using a multi-component approach to develop and deliver interventions to increase flu vaccination uptake [16]. FluCare is the first theory based multi-component intervention, designed to address individual-level barriers, by providing access to vaccines in a convenient manner and addressing complacency and confidence barriers through the provision of tailored information - see Fig. 1 [26]. The intervention is also augmented by two organisational-level strategies: performance monitoring with feedback, and £850 financial incentive for CHs with staff vaccination rates above 70% [16, 27–29]. The development of the intervention has been reported elsewhere [30].

The feasibility study for the FluCare intervention, undertaken during the 2021/2022 flu season, indicated the intervention to be deliverable and that CHs and vaccination providers (General Practice staff (GP) and Pharmacists) could be successfully recruited and were willing to participate [30]. Learning from the feasibility trial informed refinement of the intervention information materials and protocol for the cluster randomised controlled trial (cRCT) to estimate the effect of the intervention on staff vaccination rates and explore the economic impact of the intervention (e.g., cost per vaccination percentage point increase) [26].

The FluCare cRCT commenced in September 2022, with CH recruitment closing January 2023 and follow-up ending in March 2023. Despite the trial starting in September 2022, due to challenges in recruiting CHs and vaccination providers (VP’s), the first CH was not recruited until October and randomised November 2022. The primary outcome measure for the cRCT which randomised 75 CHs (38 to the control arm and 37 to the intervention arm), was the total number of staff vaccinated in a flu season divided by total number of staff employed at any point throughout that flu season. Only a small, non-significant increase in the mean % vaccination rate in intervention arm compared to intervention arm was detected after six months. However, a planned sub-analysis, including only CHs which received at least one on-site vaccination clinic, detected a significant difference (control 28.7% [*n* = 38], intervention 41.7% [*n* = 23], *p* = 0.045) [31]. Full quantitative findings on the effectiveness and cost-effectiveness of FluCare has been reported separately [30].

This paper reports findings of an embedded mixed method process evaluation of the FluCare cRCT designed to understand the implementation of the FluCare intervention and provide explanations for the observed effects in the cRCT [32]. The objectives were to:

1. Describe the intervention as delivered in terms of dose, reach and fidelity, including variations across CHs.
2. Identify contextual barriers and enablers to intervention delivery.
3. Investigate mechanisms of impact of the intervention.
4. Describe perceived effectiveness of intervention components.

## Methods

### Study design

The FluCare process evaluation was informed by the Medical Research Council’s framework on process evaluation for complex interventions [27–29]. This was a mixed methods process evaluation to understand implementation and mechanisms of impact of the FluCare intervention and to explore contextual factors influencing intervention delivery and uptake. The full protocol of the process evaluation has been reported elsewhere [32].

### The flucare intervention and cluster randomised controlled trial

The FluCare trial was a two arm, open label, definitive effectiveness and cost-effectiveness cRCT of the FluCare intervention compared to usual support, with an embedded process evaluation. The cRCT adhered to CONSORT guidelines. The design of the trial [26] was informed by a 5-arm feasibility trial conducted in 10 CHs [30]. FluCare was a behaviour change intervention designed to improve uptake of flu vaccination by staff in CHs in England. The development and components of the multi-component intervention (see Fig. 1) have been described in detail elsewhere [33].

### Study setting

Private, charity, corporate, local authority or National Health Service CHsc (both residential and nursing homes) in England providing care for older adults.

### CH and participants for embedded process evaluation

Eligibility criteria for CHs were long stay for older residents or dementia registration; self-reported staff vaccination rate < 40%; signed up to, or willing to sign up to the Department of Health and Social Care (DHSC) Capacity Tracker [34] and willing to provide weekly updates on flu vaccine status of staff and residents. The DHSC capacity tracker is a web-based tool used to gather and monitor real-time information such as COVID-19 and flu vaccination rates from care providers. All intervention CHs participated in the process evaluation, with participants including CH managers, staff and VPs. Recruitment for the process evaluation interviews begun following completion of clinic delivery in the first intervention CH and closed on 30 July 2023.

### Sample

**Fig. 2:**
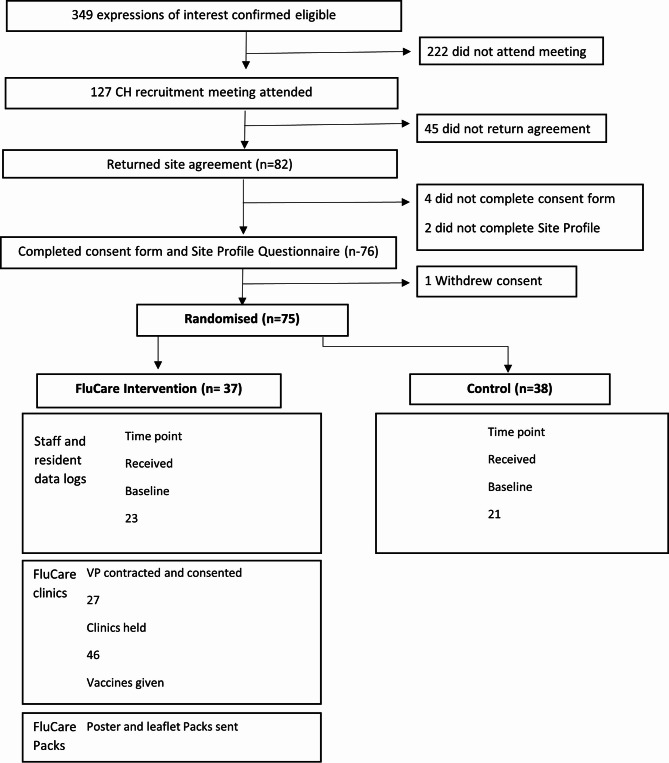
Consort diagram

Seventy-five CHs were randomised in the cRCT [38 control and 37 interventions]. A summary of recruitment and randomisation process for the cRCT is presented in the CONSORT diagram (Fig. 2). The process evaluation quantitative data was collected from all intervention CHs. Qualitative data were collected from 12 CHs (~ 30%) purposefully selected to maximise variation in contextual factors which have potential to influence implementation i.e., CH location, size, registration, and staff ethnicity (categories as defined by the Office of National Statistics) [35]. Purposively selecting a proportion of CHs for the qualitative element ensured that the work was manageable thus maintaining quality of the analysis [36]. We therefore sought to interview up to 26 CHS, 10 managers and 20 VPs, a sample size which fall within the recommended range for a large trial [36]. Participants were purposively sampled to achieve a variety of job roles and VP included those unable to deliver a flu clinic in the selected CHs. Participants were invited to participate at the end of the intervention period via email.

### Data collection and analysis

Data sources including CH Site Profile Questionnaires (SPQs), clinic logs (capturing staff demographics and flu vaccine uptake), video analytics and semi-structured interviews, were employed to examine: 1) fidelity - whether the intervention was delivered as intended (e.g., whether clinics were provided, posters/leaflets displayed and videos distributed/shown), 2) dose - the quantity of intervention implemented (e.g., no. clinics provided and how much of the video was seen by staff) and 3) reach - whether the intended audience comes into contact with the intervention, and how (e.g., whether staff were vaccinated or whether they saw and reach the posters/leaflets) [28]. Data on incentive payments and information on monitoring activities and feedback was collected from the FluCare operations study team at the end of the trial period.

- Site Profile Questionnaire (reach)
  Managers or designated administration staff in all participating CHs completed SPQs at the start and end of the data collection period. Characteristics collected included: CH type, size (i.e., number of beds, residents), number and staff role (e.g., manager, carer, kitchen staff). SPQ data were summarised to provide a contextual narrative of the CHs, highlighting any variation. The questionnaire used was specifically developed for this study and can be found in supplementary file 1.

- Clinic provider vaccination logs (fidelity, dose and reach)
  Data on clinic provision were collected from all intervention CHs. For each clinic, VPs recorded time and date of clinic delivery, duration of clinic, the number of vaccinations delivered, characteristics of each clinic attendee (irrespective of whether they got vaccinated) and any observations regarding engagement with staff during the clinic. Data from the logs were summarised to examine implementation and understand whether and how FluCare increased vaccination.

- Video analytics (fidelity, dose and reach)
  Data on video analytics were collected from all intervention CHs. Collected information included the number of clicks per CH, duration of video viewed and whether subtitles were used. Video analytics were summarised to examine implementation fidelity, reach and dose.

- Semi-structured interviews (fidelity, dose, reach and mechanisms of impact)
  Interviews were conducted virtually on MS Teams© or via telephone at the end of the intervention period by TKC, CS, AA and FA. TKC (PhD, Female), CS (PhD, Female) and AA (BsC Psychology, Female) were experienced qualitative researchers working on the FluCare trial and FA (BPharm, Male) was a PhD student. All interviewers were independent to the intervention development team and therefore declared no personal goals for doing the research. The interviewers had no prior relationship participants.
  Topic guides used were specifically developed for this study and can be found in supplementary files 2. Interviews with CHS and managers sought to understand attitudes towards flu vaccines, acceptability of the intervention, views on how the intervention was delivered and their engagement with it; and explore why the behaviour change techniques have succeeded or failed to address certain barriers. Interviews with VPs explored implementation including setting up and running clinics, and contextual factors influencing intervention implementation and engagement.
  All interviews were audio-recorded and transcribed verbatim and lasted between eight and 60 min, with an average of 35 min for managers, 15 min for staff and 21 min for VPs. Anonymised transcripts were uploaded to NVivo and analysed thematically. The analysis process, conducted by AA and FA involved initial inductive coding, followed by deductively mapping each code across process evaluation themes i.e., implementation, mechanism of actions and contextual barriers and enablers. A COREQ checklist has been provided in supplementary file 3 and a coding tree has been provided in supplementary file 4.

### Data synthesis

The focus of data synthesis was to provide an understanding of whether and how the FluCare intervention addressed barriers to flu vaccination uptake amongst CHS. It also focused on identifying and understanding variations in implementation across CH. Therefore, following initial analysis of each data item, all data sets were integrated using a triangulation approach to consider agreement, partial agreement, silence, and dissonance across the data [37, 38]. Triangulation aimed to identify and clarify causal pathways related to participant experience and implementation providing a means to explain unexpected outcomes and identify optimal intervention contexts. For example, where notable differences in clinic provision were identified from the clinic log data, qualitative data was further analysed to identify potential causal pathways for these differences.

A lay advisory group (LAG) made up of eight members of the public who had lived experience of working in a CH, having a relative in a CH or experience of social care structures supported the data synthesis. The group met throughout the study and had rolling membership of the programme management group, meaning they had a detailed understanding of the research aim, design and process. To prepare the group for input into data analysis we provided a training session in thematic analysis, led by an experienced qualitative researcher (LB). The first activity involved sharing of extracts of pseudonymised transcripts and individual LAG members coded these transcripts. These were shared and the group discussed these to develop initial themes. At this stage the researchers shared the themes they had developed and discussed areas of overlap and divergence. There was good resonance in characteristics of the themes; where there were differences these were discussed until consensus agreed, and the researchers continued to develop the analysis further. Members of the LAG reviewed all manuscripts before they are submitted.

## Results

Baseline characteristics of all intervention CHs included in the cRCT and those purposively selected for the process evaluation interviews are presented in Table 1. Apart from CH registration, baseline characteristics in the process evaluation homes were representative of those included in the study overall. Most CHs were privately owned and were registered as residential. Most staff were permanently employed in the CHs and were White/White British.

**Table 1:** Care home characteristics

Characteristics		Process evaluation care homes *N* = 12	All intervention care homes *N* = 37
Care home ownership (n (%))	Local authority	0	1 (2.7%)
	Charity	0	2 (5.4%)
	Private	12 (100%)	34 (91.9%)
Registration (n (%))	Nursing	0	0
	Residential	9 (75%)	21 (56.8%)
	Residential and nursing	3 (25%)	16 (43.2%)
Care home residents	Mean (SD) number of residents per home	37.67 (16.56)	39.4 (16.7)
	Median (IQR) number of residents per home	37 (22.5–54)	39 (27–54)
Care home staff (mean (SD))	Permanent staff	47.1 (16.6)	48.6 (19.5)
	Bank staff	3.9 (4.9)	3.7 (4.0)
	Agency staff	1.6 (3.7)	2.9 (3.1)
	Voluntary staff	2.6 (1.6)	1.1 (1.6)
Staff ethnicity, mean (SD)	White/White British	38.0 (17.3)	32.5 (18.7)
	Black African/Caribbean/Black British	2.8 (2.9)	4.2 (6.4)
	Mixed/multiple ethnic group	3.9 (8.1)	3.8 (7.1)
	Asian/Asian British	4.0 (5.0)	5.7 (10.4)
	Other Ethnic group	0.8 (1.8)	0.4 (1.3)

For anonymity, CHs included in the process evaluation interviews have been assigned a letter ID (A-L). A total of 26 interviews were conducted with CHS (see Table 2), including managers, manager & proprietor, care staff, housekeeper, cook, activities coordinator and administrator. In two of the CHs (B and G) only managers consented to interviews. In CH I, three staff consented to interviews but not the manager.

**Table 2:** Interviews CH managers and staff characteristics

Characteristics		Managers (*n* = 11)	Staff (*n* = 15)
Sex	Female	11 (100%)	13 (86.7%)
Age range	20–29	0	1
	30–39	0	2
	40–49	5	4
	50–59	2	7
	60–69	2	1
	Missing data	2	0
Ethnicity	White British	8	13
	White Other	2	1
	South Asian	0	1
	Missing data	1	0

A total of 17 interviews were conducted with VPs purposively selected to maximise variation in roles and experiences. Professions of interviewed VPs included nine pharmacists, two general practitioners, three nurses, one paramedic, one frailty practitioner and one assistant practice manager.

### Implementation of the flucare interventions components

#### Implementation flucare video (fidelity, dose and reach)

Results regarding implementation of videos in all 37 intervention CHs are presented in supplementary file 5. All intervention CHs received the promotional video which was 3 min 55 s long and had optional subtitles in four languages (English, Romanian, Polish, Hindi). The video was not accessed at all by staff in 16 out of 37 (43%) CHs. Only five staff representing five CHs viewed over three minutes of the video. The average number of clicks per CH was four (range 0–27) and the average view duration per CH was 0 min 40 s (range 0–3 min 54 s). English language subtitles were used in 19 out of the 21 (90%) CHs which had views.

#### Implementation flucare clinics (fidelity, dose and reach)

Implementation characteristics of FluCare clinics arranged by pharmacy and GP VPs in all 37 intervention CHs are presented in Table 3. 65% (24/37) of intervention CHs held at least one vaccination clinic, highlighting partial implementation of the intervention (only posters and leaflets) in the remaining 13 CHs.

A total of 48 clinics were delivered between November 2022 and March 2023, 14 of which did not vaccinate any staff. Most clinics (34/48) were delivered by pharmacy-led VPs, which tended to show greater flexibility in days the clinics were delivered (e.g., including at the weekend). Most VPs delivered one or two clinics. Variation in the number of clinics was primarily due to VP capacity (i.e. shortage of pharmacists) and late timing of flu clinics which resulted in limited availability of flu vaccines (see Sect. 3.3). A total of 146 staff were vaccinated during the FluCare clinics. After excluding 14 clinics which were held but no staff were vaccinated, the maximum total number of staff vaccinated per CH was 12 and the median (IQ) staff vaccinated per clinic was 3 [2–5].

**Table 3:** Characteristics of clinics arranged by pharmacy and GP vaccine providers

	Pharmacy-led vaccine provider	GP- led vaccine provider	Total
No. CHs			
CHs in which clinics were held	17 (46%)	7 (19%)	24 (65%)
**No. Clinics**			
Clinics held	34	14	48
Clinics (%) where at least one staff member was vaccinated	23 (68%)	11 (79%)	34 (71%)
**Vaccinated staff**			
Total staff vaccinated	101	45	146
Maximum of staff vaccinated in a clinic	11	12	12
Median (IQR) staff vaccinated per clinic	2 (0-4.8)	2 (1-3.8)	2 (0-4.3)
Median (IQR) staff vaccinated per clinic, excluding 14 clinics where zero staff vaccinated	3 (2–6)	3 (1.5–4.5)	3 (2–5)
**Day and time of clinics**			
Days of week when clinics were held	Mon-Sun	Weds-Fri	Mon-Sun
Day of week when most clinics held (#clinics)	Thursday (13)*	Wednesday (8)*	Thursday (18)*
Range of clinic start time	08:30 − 17:00	09:00–18:30	08:30 − 18:30

*Data excludes clinics vaccinating zero patients

#### Implementation of FluCare by managers: distribution of behavioural change information materials (leaflets, posters videos) and clinic organisation (fidelity)

There were notable differences in the manager reported engagement with distributing the information materials and arranging clinics. Levels of engagement of managers have been categorised as high, medium and low. This classification was done by firstly ranking the CHs by implementation (i.e., staff reports of whether posters/leaflets were displayed, video analytics, number of clinics delivered, and number of staff vaccinated) and then identifying whether this was associated with negative/positive attitudes towards being vaccinated. A sample of this mapping, with illustrative quotes can be found in Table 4 and full data mapping in Supplementary file 6.

Managers who implemented all components of the FluCare intervention (and personally engaged with the intervention to some degree) were categorised as ‘high’ engagers. Six managers were classed as ‘high engagers’ and corresponded to CHs with the highest number of staff vaccinated. These managers distributed the materials, and they were either convinced by the information provided, which for some resulted in behaviour change (getting vaccinated for the first time) or were already pro-vaccination due to personal underlying conditions. For example, Manager 001_CH-L reported creating an information corner using balloons sent by the FluCare team to get staff attention and distributed the video via staff handovers and other team meetings. The manager reported reading the materials themselves and were convinced by the materials. In preparation for the clinics the manager informed staff about the clinics and was vaccinated themselves for the first time. They then championed the vaccinations by sharing their experience of getting vaccinated to their staff. The number of staff vaccinated in this CH was 13.

Two managers were classed as ‘medium’ engagers. These managers implemented some components of the intervention without making much effort to engage staff. For example, M010-CH-C put posters and leaflets up but they themselves did not necessarily read the content. In addition, clinics were organised without proper communication to inform and engage the staff. Only one clinic was delivered in this CH despite intentions to deliver two more. The number of staff vaccinated in this CH was five.

Managers who did not fully implement all components of the intervention and held anti-vaccination views and attitudes that mirrored the very barriers that FluCare was trying to address, were classed as ‘low’ engagers. Four managers representing three CHs were grouped in this category. Some of these managers reported putting the posters up and distributing leaflets, but other staff including deputy managers did not recall ever seeing them. None of these managers appeared to have distributed the video, supported by no clicks on the video link from staff in their CHs. These managers were not so convinced about their need for the vaccine and the importance of the intervention. Staff vaccinated in these CHs ranged between 0 and 2. An example of a low engager is manager M013_CH-H who perceived that she did not need the vaccine and was concerned about the safety of vaccines. Interestingly, analysis of staff interviews from the same CH-H also highlighted similar views to that of their management (Table 7 and Supplementary file 7), possibly suggesting influence from the manager or highlighting similarity of staff attitudes within the same CH. CH-H was the only process evaluation CH where clinics were arranged but no staff got vaccinated. Interview data on manager engagement, when triangulated with data on implementation of clinics and videos (Table 3) provides explanation for variations in implementation across the CHs.

**Table 4:** Manager engagement with flucare intervention

Staff CH ID	Implementation of poster and leaflet	Implementation of video	Implementation of clinics	Attitude regarding flu vaccines for self and staff
***High engagers***: *Managers who implement and personally engage with all components of the FluCare intervention*				
**M001_CH-L**	**We just used all the materials** and created a Flu Care info point … **the balloons made it eye-catching** so that it took people’s attention… I think **I know it by heart now** because it’s literally opposite my office door … we shared some information with staff here on email and handovers.	**No. video clicks: 10** **Average view duration (mins: secs): 1:35** We shared the email [video link] **via private email**, with all the care staff. **We played after handovers** and we had some time, **we made time in the staff meetings** as well.	**No. clinics: 4** **No. staff vaccinated: 13** **We would just print a notice to signpost staff…** to let staff know where in the building…**We would pop an email out to staff** and let them know that they would be in the home at that time and **we would talk about it in handovers**.	**I’ve never had my flu vaccination which is awful because I’m the manager**…it was not because I had any reason not to have it but I just never got round to it because I’d never make the appointment…**because it was here and lots of different times**,** I was able to nip in and get it** and **then I could speak to the staff about my experience** because a lot of people are very worried about side effects and things aren’t they, so **it was nice to be able to tell them from personal experience rather than just what I’d heard.**
***Medium Engagers***: *These managers implement some components of the intervention without making much effort to engage the staff*				
**M010-CH-C**	**I can’t remember now if we got leaflets. We may well have done…. But we certainly got the posters because they went up. So**,** we had one [poster] in the staff room… We had one on our board which is outside the main office… But the main one for us is the staff room**,** because…all staff go into the staff room.**	**No. video clicks: 5** **Average view duration (mins: secs): 1:07** **The videos**,** we didn’t utilise them as much as we should have done. I don’t know how many staff did the QR code. …. The way that we got it out was the QR codes on the posters….**	**No. clinics: 1** **No. staff vaccinated: 5** **The clinics**,** they were a little bit hit and miss. So I think we had the initial one**,** which was completely my fault it wasn’t advertised for the staff…the second one I think they didn’t turn up. I don’t think they cancelled they just didn’t come in.**	
***Low engagers***: *Managers who didn’t fully implement all components of the intervention and held views that were anti-vaccination* * and whose attitudes that mirrored the very barriers that FluCare was trying to address*				
**M013-CH-H**	**We had some posters**,** yes**,** we had some brochures**,** yes**,** we had that kind of stuff…I think my staff were reading about it**,** I think I haven’t read it yet**,** to be honest.**	**No. video clicks: 0** **Average view duration (mins: secs): 00:00**	**No. Clinics: 2** **No. Staff Vaccinated: 0**	**I never had it before**,** and I really don’t want to have it… My residents have it and they get poorly afterwards anyway… I don’t really want to get anything to my body**,** you know… I’d rather just get it and get through it. Yes**,** to be honest**,** I think it’s good for the… older people like my residents… I’m nearly 50 but my immune system is quite strong So**,** yes**,** I don’t think I need it… My staff… are young people**,** they don’t really want to have it yet because they’re afraid and they’re thinking they don’t need it… Like I said we quite healthy so**,** you know**,** you can’t force anyone**

#### Performance monitoring with feedback, and financial incentives (fidelity)

Two of the 75 CHs participating recorded more than 70% of CHS receiving a flu vaccination as reported on the DHSC Capacity Tracker. Both CHs were in the intervention arm of the study and received the £850 incentive at the end of the study.

Monitoring was performed by the FluCare team, by regularly communicating with CH managers to follow-up on data completion of staff and resident logs (data collected for health economics elements of the cRCT). However, discussions with the FluCare team revealed that the intervention as rolled out did not include feedback on performance. This was due to several reasons: (1) when the intervention was designed for the feasibility study, no feedback was included due to the very short timescale of the intervention delivery (video/posters/clinics were provided and delivered within a couple of weeks); when the intervention was revisited for the cRCT trial, there was no information available on how the feedback could/should be provided; (2) CHs struggled to provide data logs on a monthly basis, such that there was at times no data upon which to provide feedback; (3) the very short time scale over which the intervention was delivered including clinics reduced opportunity for feedback. Therefore, while the study operations team attempted to monitor vaccination uptake, there was no attempt to introduce behaviour change by providing feedback on performance (e.g., highlighting how a CH was doing compared to others and discussing ways of how to improve vaccination uptake). Hence, this behaviour change element was not fully implemented.

### Mechanisms of impact

#### Staff engagement with components of the flucare intervention

To understand mechanisms of impact, staff interviews were analysed to explore engagement levels with intervention components and perceived impact of FluCare on behaviour change. Four staff categories were identified and mapped according to staff engagement with each intervention component and attitudes towards being vaccinated. A sample of this mapping, with illustrative quotes can be found in Table 5 and a full mapping in Supplementary file 7.

The first category consisted of staff who engaged with all aspects of the intervention and had the vaccine for the first time. Without the intervention these staff would not have sought the vaccine because they did not see a need for it. All staff in this group related seeing and reading the information, mainly posters and leaflets (not the videos), which for some triggered discussions with colleagues about the importance of vaccination for their role. The information then worked together with the convenience of the clinics to influence behaviour. For two staff members (S002_CH-L and S003_CH-A**)**, the primary reason for getting the vaccination was being convinced of its importance for the protection of residents. These findings indicate that, for those who have not previously had an opportunity to receive information and free flu vaccination, the FluCare intervention could influence behaviour.

The second category were staff whose behaviour was primarily influenced by accessibility/convenience of clinics. Staff in this group already had a positive attitude towards having flu vaccinations, prior to the FluCare intervention. Therefore, engagement with the FluCare materials was variable for this group and largely served as a prompt for discussions with other staff and a confirmation of prior attitudes. For these staff, motivations for having the vaccine included a perceived need to protect themselves, vulnerable residents and vulnerable family members. Although these staff reported that they probably would have sought to have the vaccine with or without FluCare, it was late in the season when the FluCare clinics were offered and they had not sought a vaccination. This suggests convenience of vaccination clinic may have been an important element of the interventions to influence their behaviour.

The third staff category did not engage with any FluCare components. These were aware of the posters/leaflets but did not read them at all. They also did not watch the video, nor engage with the clinics. The influence of non-engagement was based on prior bad experiences of flu vaccination, perceived lack of need or misunderstanding about vaccines being live. This finding suggests limited opportunity for behaviour change for those who choose not to engage with behaviour change materials.

The fourth and final category consisted of staff who were already pro-vaccination because of responsibility in their role, their own and family vulnerabilities, or their age. Some of these participants had already had the vaccine by the time the clinics were run or missed the clinic at the CH and sought it elsewhere proactively.

**Table 5:** Staff engagement with components of the flucare intervention

Staff_CH ID	Poster and leaflet	Video	Clinics	Staff attitude regarding flu vaccinations
Category 1: Staff influenced by all FluCare components i.e., either poster/leaflet or video AND clinics				
**S002_CH-L**	Yes, I did (see the posters) **It made me realise that obviously it was available**,** and I should probably do it**… **it did the job**,** it got me to have the flu vaccine**,** which I probably wouldn’t have done**.	I think so, I can’t remember now [seeing the video].	Interviewer: **If they hadn’t come into the clinic**,** do you think you would have got the flu vaccine?** **002: No**,** probably not**…**because they were there and I got the notice…** I prioritised it…**if it was up to me and I had to go and get it at the doctor’s**,** I highly doubt I probably would have managed to have gone and done it.**	I probably see it quite the same, really, they’re (covid and flu vaccine) needed for like the vulnerable, but not necessarily for everybody. But I still probably don’t fully understand how me working in a CH…I don’t understand the connection. If it’s to help me, if I was to get sick, I don’t understand why I should have it because I work in a CH.
**Category 2: Engagers with FluCare clinics**				
**S008_CH-I**	It made **it more [me] aware that the flu vaccine was available**… **the posters were quite open about when it was going to happen**.	No, I haven’t seen the video.	I **would have had the flu vaccine anyway**, but **it was just more convenient for me** to have it within my workplace	Well, I’m for having the flu vaccine. **I think the majority of people should have it because it does protect yourself** and flu is very unpleasant.
**Category 3: Staff who didn’t engage with any of the FluCare intervention components**				
**S012_CH-H**	**I didn’t read it**,** if I’m honest**, no…. Yes, I’ve just been too busy, to be fair.			I think the **flu vaccines are good for people that need it the problem is they get poorly after** the fact which obviously they need a bit more care and things like that. A bit of extra TLC but **obviously I know it’s like a live ingredient isn’t it… So it’s got part of the virus in it**,** a small amount of virus in it to build up our immunity**,** that’s what we we’ve always been taught.**
**Category 4: Staff who were already pro-vaccine and had the vaccine elsewhere**				
**S009_CH-I**	When there is a group of us in the staff room **you look at the leaflets and you start to have a discussion**… to me **if they’re giving you something that you haven’t got to pay for me personally I’ll just take i**t.	No, I haven’t seen the video, no.	I just live at the back of … my chemist is just out there. **I could have had it at work but I was off work that day but I just walked through to my chemist**,** it’s just five minutes**. **I’ve had it for the last three years nothing has happened to me**,** I haven’t had any symptoms like it’s knocked me off my feet or I’ve had to go off sick or anything like that**. I think it’s just going round and being confident in yourself that you’ve had it and then not putting a negative edge on something.	All the residents were having it and I’m thinking, what about if I get a cold…So **working in a care setting you’re going to work and then you think to yourself**,** hold on a minute**,** I can fight off this runny nose and cough and things like that but what about if I’m passing it on to these old folk** that are in their nineties.

### The role of context in flucare intervention implementation and engagement

#### Contextual barriers and enablers to delivering flucare clinics

FluCare clinic implementation was influenced by four key barriers/enablers: (1) GP and community pharmacy capacity challenges; (2) vaccine supplies related to the timing of the clinics; (3) communication between managers and vaccine providers; (4) varying levels of managerial engagement.

Where intervention homes had clinics, despite up to four onsite clinics being funded, most VP’s delivered only one to two clinics. This was due to a number of factors including pressures that GPs and pharmacies in the UK were facing during the trial period, including availability of pharmacists. Additionally, shortages in flu vaccines, especially later in the Winter season of 2022–2023, also limited delivery of FluCare clinics. Low staff vaccination uptake was also a factor.

“Like I said, later in the season it is harder to get flu vaccines so it is a little bit more difficult to have enough prepared. But, yes that was the only bit, a little bit of co-ordination that was needed.” VP_11_Pharmacist_CH36.

FluCare clinics were designed to be delivered flexibly, at different times of the day, and as walk-ins. However, due to capacity challenges, some VPs preferred pre-determining how many staff would take up the vaccine, which was not in line with FluCare intention. In addition, most GPs were not able to deliver clinics outside their working hours.

“[It] was really restrictive. We would’ve said – there are some people that only work weekends, so why couldn’t we have had one on a Saturday morning? Well, that’s an obvious no from them. People who work night shifts, can’t we have one much later in the night? That’s the sort of thing we do with staff meetings and supervisions, we work around the shifts. But obviously, with the GP, we worked to what they could do. That was a problem” M002_CH-B.

Some CH managers and VPs reported communication challenges with arranging clinics times resulting in delivery of clinics at very short notice, and without staff being aware of their availability.

“Sometimes I felt like the staff in the CH didn’t actually know that we were coming or there was a pre-booking or there wasn’t enough notice given to them. So, they weren’t prepared for it or didn’t want to help straightaway, rather than have a discussion about it” VP_11_Pharmacist_CH36.

Some VPs attributed communication challenges to a lack of engagement from the CH Manager: “So it took me a little while to set them up. The CH Manager didn’t seem to be very engaged and he said, you know, it wasn’t one of the priorities” [VP_01_Nurse_CH-C**]**. Where there was effective communication and coordination among VPs and CH managers, there was successful clinic implementation.

VP familiarity with the CH environment, as well as existing relationships with CHS, streamlined implementation.

“We already have a really, really good relationship with our GP. We’ve obviously got the enhanced GP service, and we either see the GP or the nurse practitioner at least once a week. So to incorporate [name of vaccine provider], who is the nurse practitioner, to come in and do a flu clinic, it’s kind of like, yes, she’d do the flu clinic, and then she’d just cracked on with the rest of the ward round. So, because we’ve already got that relationship and she comes in every week, yes, it was good. It just went smoothly” M002_CH-B.

#### Contextual barriers and facilitators to staff engagement with flucare clinics

Most flu clinics were delivered between January and March 2023. VPs, CH managers and staff highlighted that the lateness of delivery of FluCare clinics negatively influenced vaccination uptake, making it one of the key barriers to vaccine uptake. Starting earlier in September or October was discussed as a more effective approach.

“It was just from the study’s point of view, it started very, very late…I think if they’d been a lot earlier, so at the beginning of when people normally have their flu…but if they stay like they were this year, then definitely not. I think it was a complete waste of time. But if they were a lot earlier, then I think it would be beneficial.” M010_CH-C.

“As I said, the only downside to the service was that the FluCare study started this late. So that is the reason why we didn’t actually have a great uptake in it as well. So yes, I think maybe if it was to start at the beginning of the [flu] season, then definitely it would be much better.” VP-07-Pharmacist_CHP16.

The impact of the COVID-19 pandemic and ensuing policies was both a barrier and enabler. There was an indication that the COVID 19 mandatory legislation had a negative impact on staff willingness to get vaccination and that some staff had concerns about having both the flu and COVID vaccines at the same time.

“I know we’ve had a lot of staff that were very reluctant, and since they were forced to have the COVID vaccine, I’ve had an awful lot of staff that have turned against any sort of vaccine and are very reluctant to have, even if they used to have flu vaccines before” M005_CH-D.

However, the experience of having COVID clinics delivered in CHs, seemed to have facilitated the implementation of the flu clinics and normalised the process for staff. The COVID pandemic also played an educating role, in raising awareness of the potential impact of viral infections on themselves and residents.

“I think the whole COVID thing has made me think more about things like flu because obviously… I’m not a very sickly sort of person, I don’t get very much. So, I just assumed I had a good immunity and – but obviously nobody had immunity to COVID. So, that was a completely new thing, so we all had to rethink that side of it…so obviously then when the flu vaccine was offered, although in the past I would have always said, “No thanks, I don’t need that,” obviously that has changed my opinion now” S003_CH-A.

## Discussion

This mixed-methods process evaluation examined the implementation and mechanisms of impact of FluCare, a multi-component intervention designed to increase uptake of flu vaccinations for CHs and consisting of on-site flu vaccination clinics, behaviour change information materials, incentives and monitoring with feedback.

### Implementation (fidelity, dose and reach)

The process evaluation found that FluCare implementation varied across CHs with regards to fidelity, reach and dose. Firstly, not all intervention CHs implemented clinics or videos. Secondly, where clinics were delivered, those delivered by pharmacy providers showed greater flexibility with regards to days and times which clinics were delivered to suit staff shift patterns. In addition, the majority only delivered one or two clinics (a few delivered four). Thirdly, where videos were implemented, not all staff watched them and most staff who watched them did not watch the whole duration of it. Fourthly, the ‘feedback on performance’ behavioural change component of the intervention was not implemented in any of the intervention CHs. Generally, posters and leaflets were reported as well implemented within the sample of process evaluation CHs examined, evidenced by staff mentioning reading or seeing them.

Our findings highlight CH manager engagement as a key contributor to variations in implementation, a finding which is in line with evidence of complex interventions in CH settings [39]. Managers who were highly engaged at distributing information materials, raising awareness of the intervention to staff, and were advocates for the intervention, implemented the most clinics and had higher numbers of staff getting vaccinated. We identified low engagement and advocacy where an intervention did not align with managers personal beliefs, which suggests that employers may need to consider attitudes to vaccination and patient care when making such appointments.

The role of managers and senior staff in fostering high uptake of flu vaccination has been highlighted in research involving healthcare workers [40, 41]. The championing of vaccinations by senior staff [42] and their perceptions of importance of the vaccine [21, 43] has been associated with higher vaccination uptake in hospital trusts. Several studies have highlighted the integral role of CH managers in influencing the organisation of the CH environment [44–46]. Within the CH environment, active and participative leadership style has been associated with positive impact on changes in cultures of care and connected to innovation and implementation success [45, 46].

The FluCare intervention primarily targeted individual-level barriers and was augmented by organisational-level strategies (£850 incentive for vaccination 70% of staff and monitoring with feedback) to address evidence suggesting that staff are likely to undertake behaviours which align with organisation priorities [47, 48]. Although our findings highlight some evidence that middle managers who work closely and alongside the staff may have some influence on individual level barriers, we found no evidence indicating the influence of the organisational-level strategies. This could partly be because monitoring with feedback on performance was not fully implemented in any of the intervention CH. In addition, although monitoring (with no feedback) was performed, the lack of power position of the research team as the monitoring body, was a contributor to there being no evidence of behaviour change. Therefore, future research in this setting should consider the influence of the power position of monitoring bodies over the performance and outcome of this behaviour change strategy.

Our findings also suggests that FluCare vaccination clinics could be successfully delivered by pharmacists, GP’s, nurses and other suitably trained personnel. In addition, previous relationship between VPs and CHs has been shown to facilitate better communication and organisation of clinics. FluCare clinics were designed to be delivered flexibly, at different times and days of the week to suit staff shift patterns [26]. However, there is evidence that VP capacity challenges, limited the flexibility of delivering clinics in the evenings and weekends, and led to some providers pre-determining uptake prior to arrival rather than delivering the clinics as walk in, which the research team had anticipated would be the model for access. The qualitative data suggests community pharmacy VPs were more flexible, than GP practice providers. However, there were some CHs where clinics were delivered, but no staff got vaccinated. Therefore, in light of capacity pressures faced by GPs and pharmacies [49], it may be best to allow flexibility for VPs to have an informed sense of how many staff might attend clinics at different shift times to avoid setting up clinics where no staff get vaccinated.

Capacity issues for pharmacists and pressures faced by GP in the National Health Service made recruitment of clinic providers difficult and this was compounded by shortages in vaccine supply due to late timing of the clinics [50]. For GP providers, capacity challenges were also compounded by complex payment processes which hindered timely remuneration and reimbursement. These challenges pose important considerations for future implementation of FluCare. Since, the provision of on-site clinics is an important element of the FluCare intervention, it is important that contextual challenges are considered and addressed in designing implementation strategies that maximise engagement and improve availability of vaccine providers.

### Mechanisms of impact

Our findings suggest that the combination of behaviour change information materials (particularly posters and leaflets) and on-site clinics, might work together to influence staff behaviour towards getting vaccinated. However, the mechanism of change is complex and often dependent of managers buy-in and staff attitudes. In addition, behaviours of staff with pre-existing positive attitudes towards flu vaccination, are likely to be primarily influenced by the convenience of the clinics. Behaviour change information materials for these staff may simply serve as a means of validation of their views and attitudes, a prompt for discussion with colleagues and a reminder to get vaccinated. The fact some members of staff, despite being pro-vaccination, still had not sought vaccination outside the CH in January-March, highlights the importance of convenient accessibility to flu clinics. Due to the late timing of FluCare implementation, however, some staff who would have otherwise received the flu vaccination due to the convenience of the clinics chose not to get vaccinated. Although flu vaccine seasons runs from September to March, our findings indicate that most staff do not perceive the need and the benefit of getting vaccinated at later times in the season.

For a number of staff, the FluCare intervention did not change attitudes towards getting vaccinated. One explanation for there being no change in staff attitudes could be the group of staff who did not engage with any of the components of the intervention, especially the behaviour change information materials. This suggests there to be a group of staff where simply distributing information and delivering on-site clinics is not enough to influence behaviours. Despite a rigorous behavioural change intervention with a strong educational component co-developed with stakeholders, there was still evidence of absence of understanding of flu vaccinations and, importantly, for a few an absence of trust [51]. With some evidence showing similar attitudes towards flu vaccines amongst managers and staff within the same CH, our findings suggests that pre-existing organisational culture played an important role in deterring some in this group of staff from reading or being convinced by behaviour change materials and engaging with the clinics. This further highlights the importance of active and involved leadership in facilitating staff engagement [44–46] and suggests the need for interventions that effectively change sub-organisational cultures.

Of note was the very poor engagement of staff with the behaviour change video. Following the feasibility study, which had zero staff engagement with the videos, a change was made in the cRCT, giving flexibility to managers for tailoring video distribution according to their usual and preferred way of communication e.g., WhatsApp. Although this modification resulted in a small change in staff engagement, our findings still suggest that videos might not be the best means of delivering behaviour change information materials to CHs. The complexity of the CH setting and challenges with implementing complex interventions have been highlighted by other research [52, 53]. However, more ethnographic work needs to be done to understand the CH culture and how best to operationalise educational interventions’ components.

### Implications for practice

The results of this process evaluation foreground the important role of the CH manager in encouraging staff to take up vaccinations. The influence of the CH manager in implementation of new initiatives has been recognised in relation to the introduction of new care practices [54]. This suggests that increased time should be built into the implementation stage to work with CH managers and senior staff to explain the rationale and address behavioural issues relating to new interventions so, there is a relatively stable senior leadership team who are committed to drive innovation forward. Our findings show that there was little take up of the video irrespective of evidence that it had been distributed. As people access to Vidula media changes there may be a need to do shorter audio-visual messaging using apps used by the audience such as Tic Toc.

### Strengths and limitations

A strength of this evaluation is the consideration of all aspects of process evaluation (i.e. context, mechanisms of action and implementation – fidelity dose and reach) as recommended by current MRC guidance for the developing and evaluation of complex evaluations [40]. Another strength is the triangulation of multiple data sources which enhances the validity and reliability of our findings and minimises recall bias that could be brought on by solely relying on interview data (59). The inclusion of both quantitative data from all intervention CHs in the cRCT and qualitative data from a subsample of intervention homes, enabled us to give a full picture of the findings whilst supporting more in-depth exploration of complex mechanisms of action.

However, the lack of participation of staff in some CHsc, limited further exploration of attitudes of staff within and across CHs. In addition, the ethnic representation in the CHS we interviewed (and that of the cRCT overall), does not reflect the workforce and is a limitation to our findings. Views of staff from other ethnic backgrounds should be further examined as there may be differing barriers and enablers to engagement. The FluCare intervention included both individual and organisational level strategies to change vaccine uptake behaviour. However, although this study has thoroughly examined the mechanisms of change of individual level strategies (clinics, posters, leaflets and videos), a limitation is that it has not thoroughly examined the influence of the organisational level strategies (incentives) on behaviour change.

## Conclusion

The process evaluation of the FluCare cRCT highlights variations in implementing the FluCare intervention with regards to fidelity, dose and reach. The findings also highlight the key role of manager engagement and their attitudes in facilitating implementation. The findings show that the multi-component of intervention, can change staff attitudes and behaviours towards getting vaccinated but the mechanisms of change are complex. Pre-existing sub-organisational culture was a key barrier for staff who did not engage with any component of the intervention. More work is needed to examine the influence of existing sub-organisational cultures and leadership styles in staff engagement with behaviour change interventions to design appropriate strategies. Contextual factors such timing of intervention delivery were important barriers for staff who did not engage with the clinic component of the intervention. With the intervention being delivered so late in the season, however, it is difficult to ascertain the true magnitude of these contextual factors on overall vaccination rate. A trial which provides onsite access to clinics from the start of the flu season will provide a better sense of the overall remaining resistance to vaccination by CHS.

## Supplementary Information

Supplementary Material 1.

Supplementary Material 2.

Supplementary Material 3.

Supplementary Material 4.

Supplementary Material 5.

Supplementary Material 6.

Supplementary Material 7.

## Data Availability

FluCare data and materials are available for secondary research purposes. In the first instance, requests should be directed to Dr Amrish Patel (amrish.patel@uea.ac.uk). Release of data may be subject to completion of a data sharing agreement.

## Abbreviations

CH: Care Home
CHS: Care home staff
GP: General Practice
cRCT: Cluster randomised controlled trial
VP: Vaccination provider
