# Supplementary material for: Process evaluation of the flucare cluster randomised controlled trial: assessing the implementation of a behaviour change intervention to increase influenza vaccination uptake among care home staff in England
Source: BMC Health Serv Res. 2025 Aug 21;25:1118. doi: 10.1186/s12913-025-13298-0 (PMC12369172; doi:10.1186/s12913-025-13298-0)
Supplement: Supplementary file 1 — Supplementary Material 1. [file 12913_2025_13298_MOESM1_ESM.pdf]

FluCare: Estimating the effectiveness and cost-effectiveness of a complex intervention to increase care home staff influenza vaccination rates.

Site Profile Questionnaire

This questionnaire is designed to help us think about how FluCare could be implemented within your care home. We would therefore be grateful if you could answer the following questions to help us understand your team. Please provide as much detail as possible.

If any of the questions are unclear, please ask a member of the research team for clarification. If you are not certain of your answer, please provide a rough estimate.

Date ☐☐ / ☐☐ / ☐☐

Care Home and Residents

1. What is the name and postcode of your care home?

Care Home name ________________

Post Code ________________

2. What is the ownership of your care home?

Private ☐ Charity ☐ Local Authority ☐ NHS Owned ☐

3. How many beds does the care home currently have? ☐
(Please enter a number between 1 and 399)

4. What is registration of your care home?

Residential ☐ Nursing ☐ Both residential and nursing ☐

5. How many residents currently live in the care home on a permanent basis? ☐
(Please enter a number between 1 and 399)

Care Home Staff

6. Is your Care Home signed up to the capacity tracker?

Yes ☐ No ☐

If 'No', when do you intend to sign up to the capacity tracker?

Date: ☐☐ / ☐☐ / ☐☐

7. Roughly how many staff are currently directly employed by the care home?
(If there are no staff in a category, please enter 0 (zero))

	Permanent Staff	Bank Staff
Management	☐	☐
Admin Staff	☐	☐
Direct Care Team – Registered Nurses	☐	☐
Direct Care Team – Others:	☐	☐
Cleaning Staff	☐	☐
Kitchen Staff	☐	☐
Activities co-ordinator	☐	☐
Maintenance	☐	☐
Mixed roles<br>*(Please provide details in the box below)*	☐	☐
Other<br>*(Please provide details in the box below)*	☐	☐

Please provide details for 'Mixed roles'

Please provide details for 'Other' (including anyone else that regularly works in the care home)

8. Are there currently any agency or voluntary staff working in the care home?

Yes ☐ No ☐

If yes, roughly how many agency/voluntary staff currently work in the care home?
(Please provide a number between 1 and 399 - If there are no staff in a category, please enter 0 (zero))

Agency ☐ Voluntary ☐

What roles do agency and voluntary staff have in the care home? *(Please provide number in appropriate column. If there are no staff in a category, please enter 0 (zero))*

	Agency Staff	Voluntary Staff
Management	☐	☐
Admin Staff	☐	☐
Direct Care Team	☐	☐
Cleaning Staff	☐	☐
Kitchen Staff	☐	☐
Activities co-ordinator	☐	☐
Maintenance	☐	☐
Mixed roles	☐	☐
Other	☐	☐

Please provide details for 'Mixed roles'

Please provide details for 'Other'

☐

9. Do all staff currently have contact with the care home residents? (if no, please provide details)

Yes ☐ No ☐

Specify: what roles do not have direct contact

☐

10. Roughly how many employed staff are?
(If there are no staff in a category, please enter 0 (zero))

Full time ☐ Part time ☐

11. What working shift patterns are available in your care home? *(Tick as many as apply)*

12-hour shifts (e.g., 8am-8pm or 8pm-8am)	☐	6-hour shifts (e.g., 2pm-8pm)	☐
Weekday only	☐	Weekend only	☐
Day shifts only	☐	Night shifts only	☐
Other	☐	(Please provide details in the box below)	

Specify 'Other shift pattern(s)'

☐

12. Roughly how many of your employed staff define themselves as? (include permanent and bank staff)
(If there are no staff in a category, please enter 0 (zero))

White or White British ☐

Black African/Caribbean/ Black British ☐

Mixed/Multiple ethnic group ☐

Other ethnic group ☐
(Please provide details in the box below)

Asian/Asian British ☐

Not known ☐

Please provide details for 'Other ethnic group'

13. **Roughly how many of your employed staff define themselves as? (include permanent and bank staff)**
(Please insert number, if there are no staff in a category, please enter 0 (zero))

Woman ☐ Man ☐ Other* ☐ Don't Know ☐

(*Other to record transwoman, transman, non-binary/genderqueer/agender/gender fluid)

Flu Vaccinations

14. **How do your staff receive their flu vaccinations?** *(Tick as many as apply)*

Staff receive flu vaccination in the care home by GP ☐

Staff receive flu vaccine in their GP practice ☐

Staff receive flu vaccination in the care home by Pharmacist ☐

Staff receive flu vaccine in community pharmacy ☐

Don't know ☐

Other (please provide details) ☐

Please provide details for 'Other':

15. **What do the care home use to communicate information about flu vaccinations?** *(Tick as many as apply)*

Mandatory training	☐	Meetings	☐	Posters	☐
Videos	☐	Leaflets	☐	Emails	☐
Other (please provide details)	☐	None of the above	☐		

Please provide details for 'Other method(s)'

16. **How does management communicate general information directly to staff (i.e. news updates, training opportunities, shift schedules)** *(Tick as many as apply)*

Email	☐	Paper handouts	☐
WhatsApp/Messaging App	☐	Meetings	☐
Online platform	☐	Other (please provide details)	☐
Text	☐		
Phone call	☐		

Please provide details for 'Other method(s)'

17. **Do staff or the care home currently receive any incentives to get the flu vaccine?**

Yes ☐ No ☐

If yes, please provide brief details:

Care Home policies and procedures

18. How do you inform staff about infection control policies or protocol changes in the care home? *(Tick as many as apply)*

Email	☐	Paper handouts	☐
WhatsApp/Messaging App	☐	Meetings	☐
Online platform	☐	Other (please provide details)	☐
Text	☐		
Phone call	☐		

Please provide details for 'Other method(s)'

19. Which vaccines (if any) does your care home currently have policies in place for? (e.g., Covid, Hepatitis B)

Please provide details:

20. Does the care home have any protocols in place for when staff are sick with the flu or other infections?

Yes ☐ No ☐

If yes, please provide brief details:

21. Do you currently have a system to collect and record information on staff flu vaccination status?

Yes ☐ No ☐

If 'Yes', please describe

22. **Has there been any protocol or guidance change in the care home with regard to staff being personally vaccinated against flu during the last year?**

23. **Are you aware of any upcoming changes/events that might impact the delivery of FluCare within your team (e.g., staff changes, funding changes, organisational changes, restructuring, new policies or initiatives)?** [This question for first SPQ only; remove for end of study]

Yes ☐ No ☐

If yes, please provide brief details:

24. **Are there any other comments that you would like to share about FluCare?**

Thank you for completing this information!
