# Supplementary material for: Process evaluation of the flucare cluster randomised controlled trial: assessing the implementation of a behaviour change intervention to increase influenza vaccination uptake among care home staff in England
Source: BMC Health Serv Res. 2025 Aug 21;25:1118. doi: 10.1186/s12913-025-13298-0 (PMC12369172; doi:10.1186/s12913-025-13298-0)
Supplement: Supplementary file 2 — Supplementary Material 2. [file 12913_2025_13298_MOESM2_ESM.docx]

# **FluCare (Phase 3) Process Evaluation Topic Guide for Pharmacists or GP practice staff**

As the purpose of the interview is to encourage a conversation in which the participant can feel confident in expressing their own views, the following topic guides are indicative. This means that the interviewer may adapt it to suit the conversation style and preferences of the participant.

|  | Introduction.   - 1. **Introduce yourself**   2. **Explain the purpose of the research** | You have been delivering a new service in which care home staff can opt to receive Flu vaccinations within their care home, workplace. We now want to explore your experiences of delivering this service. |
| --- | --- | --- |
|  | The digital recorder  1. **Stress confidentiality** 2. **Set ground rules** | I would like to highlight the confidentiality of everything you tell me, and specifically that:   - *you won’t be identified individually in any report* - *all information will be anonymised* - *we will not tell anyone else including your employer organisation, what you tell us as an individual.* - *likewise, no other individual e.g., pharmacist, care home staff, resident will be identifiable in any report.*   *We do need to remind you, however, that* if you do disclose anything which might identify a risk to yourself or to others, or a personal or professional offence, this would be shared with the relevant responsible authority. However, we would tell you if we thought this were the case.   - All your views are of value to us. There are no right or wrong answers, - Please ask me to clarify if the question isn’t clear. - We remind you not to share any personal or patient identifying information during this interview |
|  | Ask if there are any questions | |
|  | **Confirm consent** | |

|  | **Stem question for Pharmacists** | **Probes / follow ups** |
| --- | --- | --- |
|  | Intervention |  |
| 1. | Overall, what are your thoughts on the FluCare study? | - Views on delivering vaccinations in CH setting for staff |
| 2. | How has the FluCare intervention impacted on your workload? | - (Negatively / positively) - Time including time out of pharmacy/GP practice - Visit frequency - Access to vaccinations/pharmacy supplies for clinic - Record keeping |
| 3. | In what ways does FluCare fit with your contracted pharmacist or practice work? | - Local vaccine initiatives - Reimbursement and payment processes - Workplace targets |
|  | Clinic Implementation |  |
| 4. | How did you organise running the flu vaccination clinics in the care home? | - Communication with CH - Location of clinics - Time taken to set up and close down - Communication with staff during clinics - Support from manager/team - Ease of vaccinating on site |
| 5. | What made it easy for you to run the FluCare clinics in the care home?  What made it difficult to run FluCare clinics in the care home?  What could have made it easier to run the clinics? | - Liaison with the care home manager - The Site Initiation Visit - Communication with the FluCare team - Instructions provided by the FluCare team - Working with others (e.g., manager, staff, gp/pharmacy colleagues, researchers) |
| 6. | Were there reasons why staff who attended the clinic did not receive a vaccination? | - Reasons why if not (e.g., waiting times) |
| 7. | How has the new service affected your relationship with the care home managers and staff? | - New contributions - New problems |
| 8. | Have there been any communication issues during your time delivering the FluCare clinics?  If so, what were they? | - Care home manager - Care Home staff - Pharmacist or practice colleagues - Residents - Relatives - FluCare team |

|  | Acceptability |  |
| --- | --- | --- |
| 9. | How well do you think FluCare clinics worked in the care home? | - Positives - Negatives |
| 10. | How could the FluCare clinic have been improved? | - Communication - Planning and organisation |
| 11. | Would you like the vaccination service to continue in the care home you currently support? Why/why not? | - In its current from - In a revised form - Not at all |
| 12. | Any final comments? |  |
| 13. | Thank you for taking part in this interview | |
