# Supplementary material for: Process evaluation of the flucare cluster randomised controlled trial: assessing the implementation of a behaviour change intervention to increase influenza vaccination uptake among care home staff in England
Source: BMC Health Serv Res. 2025 Aug 21;25:1118. doi: 10.1186/s12913-025-13298-0 (PMC12369172; doi:10.1186/s12913-025-13298-0)
Supplement: Supplementary file 4 — Supplementary Material 4. [file 12913_2025_13298_MOESM4_ESM.pdf]

Codes

Name	Files	
(Manager) Attitudes and Beliefs	9	
(Staff) Attitudes and Beliefs	27	
attitudes towards incentives	12	
CONTEXTUAL FACTORS	3	
Barriers	8	17
Covid-19 Cons	23	59
Materials characteristcis	1	1
Timing	10	22
Facilitators	4	6
Carehome characteristics	2	7
Covid-19 Pros	9	15
Existing efforts to encourage or track vaccinations	12	
IMPLEMENTATION	10	
Flu clinic provision details	9	39
How FluCare was communicated and supported b	17	38
LeafPost set up (Manager)	9	17
Staff awareness of materials	11	21
Video distribution (Manager)	8	9
Manager Flucare Engagement Efforts and Attitudes	0	
High	4	27
Low	4	18
Medium	3	22
MECHANISMS OF IMPACT	10	
Improved Understanding (+Staff engagement)	20	52
did staff talk about it	11	18
Perception of Flu Care overall	12	24

Codes

Name	(link icon)	Files
— — Staff Perception of Flu Care	13	19
— Perception of Flu Clinic	21	50
— — responses to clinic	15	35
— — — getting vaxxed (outside of Flu Care)	6	11
— Perception of Materials	22	48
POTENTIAL IMPROVEMENTS		19
response to questionnaire		5
Staff FluCare engagement		0
TDF		0
