# Supplementary material for: Process evaluation of the flucare cluster randomised controlled trial: assessing the implementation of a behaviour change intervention to increase influenza vaccination uptake among care home staff in England
Source: BMC Health Serv Res. 2025 Aug 21;25:1118. doi: 10.1186/s12913-025-13298-0 (PMC12369172; doi:10.1186/s12913-025-13298-0)
Supplement: Supplementary file 5 — Supplementary Material 5. [file 12913_2025_13298_MOESM5_ESM.docx]

**Access to the FluCare promotional video**

| **Care Home ID** | **Total no. of video clicks** | **Avg. view duration (mins: seconds)** | **Subtitles used  (% of clicks using subtitle)** |
| --- | --- | --- | --- |
| 01 | 0 | 00:00 | Video not viewed |
| 02 | 0 | 00:00 | Video not viewed |
| 03 | 0 | 00:00 | Video not viewed |
| 04 | 0 | 00:00 | Video not viewed |
| 05 | 0 | 00:00 | Video not viewed |
| 06 | 0 | 00:00 | Video not viewed |
| 07 | 0 | 00:00 | Video not viewed |
| 08 | 0 | 00:00 | Video not viewed |
| 09 | 0 | 00:00 | Video not viewed |
| 10 | 0 | 00:00 | Video not viewed |
| 11 | 0 | 00:00 | Video not viewed |
| 12 | 0 | 00:00 | Video not viewed |
| 13 (A*) | 0 | 00:00 | Video not viewed |
| 14 (G) | 0 | 00:00 | Video not viewed |
| 15 (F) | 0 | 00:00 | Video not viewed |
| 16 (H) | 0 | 00:00 | Video not viewed |
| 17 (D) | 1 | 00:04 | English: 100% |
| 18 | 2 | 00:13 | English: 100% |
| 19 (J) | 2 | 00:32 | None: 100% |
| 20 | 2 | 00:40 | English: 50%; None: 50% |
| 21 | 27 | 00:49 | English: 89%; None: 11% |
| 22 | 20 | 00:53 | English: 5%; None: 95% |
| 23 | 2 | 01:00 | English: 100% |
| 24 (C) | 5 | 01:07 | English: 100% |
| 25 | 11 | 01:27 | English: 64%; None: 36% |
| 26 | 7 | 01:31 | Polish: 14%; None: 86% |
| 27 (K) | 12 | 01:34 | English: 83%; None: 17% |
| 28 (L) | 10 | 01:35 | English: 10%; None: 90% |
| 29 | 20 | 01:49 | None: 100% |
| 30 | 4 | 01:52 | English: 75%; None: 25% |
| 31 (E) | 11 | 02:08 | English: 91%; None: 9% |
| 32 (B) | 4 | 02:09 | English: 75%; None: 25% |
| 33 | 1 | 03:41 | English: 100% |
| 34 | 1 | 03:51 | English: 100% |
| 35 (I) | 1 | 03:54 | English: 100% |
| 36 | 1 | 03:54 | English: 100% |
| 37 | 1 | 03:54 | English: 100% |

*Process evaluation care homes represented by letters A-L
