# Supplementary material for: Process evaluation of the flucare cluster randomised controlled trial: assessing the implementation of a behaviour change intervention to increase influenza vaccination uptake among care home staff in England
Source: BMC Health Serv Res. 2025 Aug 21;25:1118. doi: 10.1186/s12913-025-13298-0 (PMC12369172; doi:10.1186/s12913-025-13298-0)
Supplement: Supplementary file 6 — Supplementary Material 6. [file 12913_2025_13298_MOESM6_ESM.docx]

**Category 1:** **High engagers**

**Definition**: Managers who implemented all components of the FluCare intervention and personally engaged with the intervention.

| **Staff ID-Care Home ID** | **Poster and leaflet** | **Video** | **Clinics** | **Managers attitude regarding flu vaccinations  for self and/or staff** | **Views about the intervention** |
| --- | --- | --- | --- | --- | --- |
| **M001_CH-L** | We had a message board, so we just used all the materials and **created like a Flu Care info point really**. I think I know it by heart now because it’s literally opposite my office door…we have had meetings in my office so that’s an area that staff are, and because staff are constantly obviously around the manager’s office… obviously we shared some information with staff here on email…**we spoke about it in handovers and in department meetings**...Like I said the balloons made it eye-catching, so as soon as the balloons went up people were asking about it and engaging with it. So I think for us it was just about having a centralised piece of information…. And it made it more of a topic of discussion.  Clinic posters: **We just put notices up to signpost staff** … more to let staff know where in the building because it might not be in the same place every time | **No. clicks: 10**  **Average view duration: 01:35**  **We shared [video link] via email, private email**, with all the care staff. **In staff meetings we also played the video and we discussed amongst ourselves** really. So it’s our handovers tend to be quite informal, staff also have a laugh and a joke and talk about things and it’s quite a good informal way to talk about people’s worries or concerns and what they’re worried about | **No. clinics delivered: 4**  **No. staff vaccinated: 13**  Well they [vaccination providers] would just call up and notify us that they had space certain days and times, **we would pop an email out to staff and let them know that they would be in the home at that time and we would talk about it in handovers**. And when they got here we would provide them with space, they would set up, **we would [name of other staff member] bless her rather than myself, would be just prompting staff and just reminding them that they were here and if they wanted to go and see them they could go and have their vaccination.**  It definitely I think had an effect on who would take up the vaccine, the fact that we had several different dates and times where somebody was coming into the home to provide vaccines rather than it just being if you’re not here on this date and time if we’re going to get a vaccination in the home at all. | I mean it’s a bit awful really and I probably shouldn’t say it, but **I’ve never had my flu vaccination which is awful because I’m the manager**, but it was more something that I never got round to…so it was not because I had any reason not to have it but I just never got round to it because I’d never make the appointment, I’d never go and have it and **because it [FluCare clinics] was here and lots of different times, I was able to nip in and get it and then I could speak to the staff about my experience** because a lot of people are very worried about side effects and things aren’t they, so it was nice to be able to tell them from personal experience rather than just what I’d heard.  . | So, **the information that we received was really good, easily accessible for staff** and residents… I think the information was **concise and it was in a way that we would speak to each other**... It was the things that people actually are concerned about and think about. **So I particularly liked… where it was talking about the common questions that people would ask** like ‘Is this going to affect me? Do I need it?’ Because I had heard people speaking and saying, ‘Oh God I had my flu jab and it was awful and I felt terrible, it was worse than flu,’ and all those sort of things, so that was quite reassuring  . |
| **M012_CH-K** | We did our own little display to support the – you know, to get the staff on board. And it did actually – I think if we did this year on year, I think more people would be encouraged to do it. But a lot of people did have the [vaccination] – more than [previously]. It wasn’t a massive increase, but it was a lot more than it would have been.  When we got the information, it was shown more in our daily morning meetings. So the idea was the team leaders would go and speak to the staff, and then in the staff meetings, we would discuss it.  We would also [use] our staff WhatsApp groups – because that’s the easiest way to get to them, is through that. And it’s such a modern cliché, but that’s how we got to them. So we used some of the information and put it into that – so they were reading it. And a lot of them got really interested in that way. But I think the most positive was the display, because you could just walk past and you could see them being interested in that. | **No. clicks: 12**  **Average view duration: 01:34**  So the videos were – and we did through our daily morning meetings. And staff were given an opportunity through the links. | **No. clinics delivered: 2**  **No. staff vaccinated: 12**  I went in first, and I put a picture in all the staff chats, which I do, to say I’ve done it…. And then I try and encourage everyone. And everyone was coming down to have it done. | I went in first, and I put a picture in all the staff chats, which I do, to say I’ve done it…. And then I try and encourage everyone. And everyone was coming down to have it done. And I think now, what I think – really I should have about four vaccines, because then more will have it. But I think if we can get – encourage them. Because people will – I think this is what was – I think you need to say it to them in layman’s terms that – like, just before Christmas everyone was getting really poorly with different flus, different – and even I was, because I missed my flu jab as well. | I always have the flu jab, so I’m more pro vaccinations. |
| **M003_CH-E** | So obviously, the posters, they was dotted around the home and put in the staffroom on the notice boards at the entrance, they were shared with relatives and staff via email. We also shared, talked about [FluCare at] meetings, staff meetings that I had, and obviously the justification behind it, and the reason why it was so important, just increasing people’s knowledge. The leaflets were left in significant places. It were all relevant and all went around it. | **No. clicks: 11**  **Average view duration: 02:08**  They were all sent the link via email, the staff, to access the videos… and there were a couple of staff that all watched it together on one form. Some of them did it on shift. Some of them did it with the activity coordinator. Some even did it in here with me on my computer, when we had a flash meeting, and I showed them that. | **No. clinics delivered: 1**  **No. staff vaccinated: 8**  So I have obviously different meetings. I have daily flash meetings, which I have with the senior team, who then relay down to the care team, but I had a full staff meeting, which I have usually every couple of months, in readiness for this, and attendance of that was quite high. And I explained what we were doing and the idea behind it, and like I say, I also have quite a few ethnic minority staff within the home, recently over from India in the last couple of years or so, and I do believe a couple of them actually went and had the flu vaccine. Yes, they did. | If they have it, if they don’t, I’d never pressure them into having it. I do believe, quite firmly, it’s down to the individual, but I do believe that education is obviously the first point, because people say no to things they don’t understand. But once I know that we’ve done everything to empower them into having all the information and the access to things, and then they decide not to anyway, then that is their decision and I respect that. I won’t force them to do anything else after that. | It were all relevant and all went around it. |
| **M004_CH-J** | I put some in our entrance hall, so when staff were signing in they saw the smaller posters which is quite good and also then family and other relatives would see and it gave them reassurance that we’re actually doing something to protect their family. And I also put some in the staff toilets which was good because you’re sitting there, you’re not doing anything else except looking at a poster.  No [I did not use any large posters] because our care home is quite a homely care home, it’s a very old building, it’s quite small and it would have overlapped into the residents’ area. | **No. clicks: 2**  **Average view duration: 00:32**  I sent links for the video through our staff WhatsApp site, so we did it that way. To be honest I can’t even remember the video, it sounds terrible doesn’t it? Nobody said anything about the video, ‘We watched it’ but that was kind of it. | **No. clinics delivered: 2**  **No. staff vaccinated: 8**  We have WhatsApp groups so I would put in clinic times, make sure you’re there, be on time, kept reminding them and reminding them, put in little pictures of flu and all sorts into the WhatsApp but just as a reminder all the time just to push it and push it.  Well generally if I put something in they put a thumbs up to say I’ve recognised you’ve put something in the group so I didn’t get much of a feedback from the WhatsApp, but the fact that they turned up, you know is a good thing. | They have it if they want it, you know, it’s your own decision … No I think the NHS have promotions going to say it’s a good idea to get your flu jab, blah, blah, blah, and the owner of the homes, he did work in the NHS as a nurse so he is quite hot on people going to get their injections and reminding me but then I always, I think probably because I’ve got an underlying health condition, I know the flu jab is important and I have to say we did have a lot of terrible flu this year within the home and the staff were coughing and sneezing and I didn’t get anything. So yes, so it’s important for me. | It’s just the flu jab, it wasn’t anything new. I think if it was something new they’d be ‘What’s that for?’ But everybody knows of the flu jab" |
| **M005_CH-D** | So the posters, I just put up around the home. We’re only a little home, so we didn’t use them all. Yes, so we put them by the back door, which is the main staff entrance, on both sides of the door that they use for going out for breaks and coming in. And then we had them in the main office, where they tended to go into a lot. We didn’t have them around the communal areas with the residents, because we tend to have those sorts of things for the residents. And we had one by the front door, so each time we were letting in visitors, it was there in front of them. | **No. clicks: 1**  **Average view duration: 00:04**  So we did the staff meeting and I sent the video out at the same time. So, we talked about the Flu Care, and then as part of the minutes, I sent them the video as well, so that it was relevant and not just an email on its own, sort of thing, so at least they knew what it was all about. | **No. clinics delivered: 2**  **No. staff vaccinated: 7**  Well, we had two clinics. I’d already gone through everybody and spoken to everybody individually anyway about the vaccine, saying, “We’re going to arrange this so you don’t have to go anywhere. There are these places you can go. And we went through everything, and I had my note of who would, who was possible, and who definitely. And I knew with the definite nos, there was no way I was going to – I wasn’t even really going to properly try because I didn’t want them to lose their trust in me as well. But there were people that were umming and ahing, and I arranged it on a day that the ones that [they] were in work because I knew that they were on the border, but I’m not going to go and get it elsewhere, but if it’s offered here, then I might do it. So, I arranged it for when they were in work. | I’ve always had it because of my asthma | I’ve always had it because of my asthma |
| **M002 _CH-B** | I used everything. Poster up, a couple of leaflets on the tables where they actually have to sign the fire book as well, because that’s where we tend to – like for example, we’ve got a garden party on Monday for the coronation, and it’s on the fire – where they sign the fire book for in and out, near the clock-in machines, that’s where we leave stuff, you know like, “Give us a donation for the raffle,” and “don’t forget to turn up for the garden party.” So it is an area that we do use, but we’ve certainly never done it like we did for the flu thing. | **No. clicks: 4**  **Average view duration: 02:09**  We watched the video at handovers, and they were all given the link to watch it. So, whether they did or not, I don’t [know]. They’re really not the most enthusiastic bunch to do anything over and above on those sorts of occasions. But it was prompted and reminded at handover for weeks and weeks and weeks, and we still just didn’t get through. Blooming COVID, that’s all I can say, is blooming COVID. | **No. clinics delivered: 1**  **No. staff vaccinated: 5**  We have, it’s like a WhatsApp for all the staff. It’s called Silo. It’s actually an NHS data protected messaging system, and everything gets deleted after 30 days, for confidentiality. So not only on handovers, I’m putting on silo, “Don’t forget, flu clinic,” on whatever day it was at whatever time it was. “Anybody can’t come and wants another date, let me know, we’ll see what we can arrange.” | So I think probably by the end, they [staff] probably thought I was really badgering them, but I kept saying, “I’ll be first in the queue. I’ll be the example. I’m not telling you to do something I’m not going to do myself.” | I actually think it was a very positive intervention. And I was actually fully behind it. I was the one with the balloons and the posters.. and telling everybody to do it, and arranging the clinics. And I just think, for us, and probably for many others, it just came at the wrong time, and everybody was just COVID out. They’re just so fed up, and it’s like with this next round of COVID spring boosters, nobody wants them. |

**Category 2: Medium engager**

**Definition**: Managers who do not fully implement all components of the intervention.

| **Staff ID-Care Home ID** | **Poster and leaflet** | **Video** | **Clinics** | **Staff attitude regarding flu vaccinations and other staff** | **Views about the intervention** |
| --- | --- | --- | --- | --- | --- |
| **M010_CH-C** | I can’t remember now if we got leaflets. We may well have done. It was quite a while ago. But we certainly got the posters, because they went up. Yes, I can’t remember getting leaflets though.  So we had one [poster] in the staff room. So our staff room, every single member of staff goes into the staff room. And then I think we had one on our board which is outside the main office, which is what the visitors see. So this is our visitors’ entrance. So the visitors would have seen it as well as staff using it. But the main one for us is the staff room, because that’s where – all staff go into the staff room.  It was all discussed all in depth in the management meetings in terms of that. So yes, all staff, depending on what their role within the home is, have varying degrees of being aware of what the study was about. | **No. clicks: 5**  **Average view duration: 01:07**  The videos, we didn’t utilise them as much as we should have done. I don’t know how many staff did the QR code. I couldn’t tell you that. So obviously the way that we got it out was the QR codes on the posters. Because again, that link – I think we as a home, because we’re quite busy, we needed to have maybe done sessions within our home to play the videos. So I think from our point of view, we’ve got learning from it as well. But taking part in these things, we need to be slightly more involved than what we did. So I think there’s yes, learning for us as well. | **No. clinics delivered: 1**  **No. staff vaccinated: 5**  The clinics, they were a little bit hit and miss. We had the initial one, which was completely my fault it wasn’t advertised for the staff. The second one we had done that again but a bit more not last minute. And then they obviously didn’t turn up. I don’t think they cancelled. And then the [third] one, I think because the majority of our staff had already had their vaccinations…it was too late. But I think going forward, if we do it again, then yes, we need to do a bit more. like posters up to say, “Flu clinic on this date. Come on in.” That sort of thing would be – from our point of view, would be good to do. |  | The education of the staff, we have quite a varied mixture of ages and ability within our care home. So I think one thing that could have been a bit better was – obviously we had the posters and we had the QR code where they could go on and download the videos and things. But maybe you could look at other ways that you could educate staff other than a QR code and a poster. Just an idea, only because some of our older staff, they wouldn’t be looking at QR codes and that sort of thing. Because it would just go over their head, and they’d be, “Oh, I don’t want to be dealing with that.” But that’s from our personal point of view. |
| **M007_CH-A** | I downloaded them and made copies, I put them in the staff room on the notice board, down the hallway, just to say we were committed to this, but I think I spoke about it with the staff. | **No. clicks: 0**  **Average view duration: 00:00**  I mean it was nearly six months ago, you want me to have a brain for six months ago, really? | **No. clinics delivered: 1**  **No. staff vaccinated: 2** | But of course I have the flu jabs because I’m on the vulnerable because I am diabetic, so I just get them routinely every year, just go along, shove it in and out I come. |  |

**Category 3: Low engagers**

**Definition**: Managers who did not fully implement all components of the intervention and held views that were anti-vaccination and whose attitudes mirrored the very barriers that FluCare was trying to address

| **Staff ID-Care Home ID** | **Poster and leaflet** | **Video** | **Clinics** | **Staff attitude regarding flu vaccinations** | **Views about the FluCare project** |
| --- | --- | --- | --- | --- | --- |
| **M011_CH-G** | Well, to be honest I had a lead person who was sort of championing it. And then she went off sick for some time, so she may have received them. I don’t recall ever seeing them. I have seen posters batted about. | **No. clicks: 0**  **Average view duration:00:00**  I haven’t seen a video. So I couldn’t tell you about that, to be quite honest." | **No. clinics delivered: 0**  **No. staff vaccinated: 0**  The key for me is that we have those clinics in the home. And solely that, really. I think had we have had the clinics in the home, we would have had a massive [uptake? 00:19:08]. Irregardless of the fact that they’d all had the Covid vaccinations, and perhaps they were still thinking, “Well, flu’s vanished. Now it’s Covid,” I still think they would have taken up the opportunity to have the flu vaccination. I mean, I’ve not had it this year. I would have probably have had it definitely if it had been in the home. |  | Absolutely pointless to be quite honest with you. Well, we’ve had nothing. We’ve had no feedback. It’s just been a pointless exercise, to be quite honest. |
| **M006_CH-H** | It was easy to take a picture of the poster and put it on WhatsApp, it was easy to communicate.  I was just talking to the staff during our team meeting. So I did it during a team meeting because otherwise there was no way I was going to get people together. | **No. clicks: 0**  **Average view duration: 00:00**  No, no I didn’t ask did you see the video and what do you think, so I can’t give any. | **No. clinics delivered: 2**  **No. staff vaccinated: 0**  **I did not do any effort to change their minds because obviously that wasn’t part of the system...  I just facilitated.** So I told via the WhatsApp group again that the pharmacist would come for the clinic, would you let me know if you’re thinking about coming.  So the days that the clinics were there, the two days, I would mention in the morning in the WhatsApp group that the clinic was there again and then of course people would know that it was there at the moment that they would have a changeover and the set up was a gentleman in the clinic doing the talking. He was in the same area as where we do the handover, so it was pretty unavoidable.  It was unavoidable. I needed to make it rather than making it accessible, I needed to make it unavoidable. They were trying to avoid everything..**..** | Interviewer: And when you say that there is kind of no proof that it works, could you just explain a little bit about that?  **I don’t think from anything of the research I’ve looked at that we know, and I think the research should show that, that we know that people make less people in a care home ill as a result of having had a flu vaccination.** So the reason you would do a flu vaccination is so less people in the care home will get ill to the extent that they either have to use an NHS service or they die. So there’s two reasons why you would want a flu vaccination. Neither of those two have been proven to have an effect on this age group as well.  Interviewer: So [you mentioned] how incentives [money] to get vaccinated could be unethical, could you just elaborate a little bit about that where you can?  Because I think you’re actually bribing people to do something with their bodies that they don’t want to do. When they believe that it’s not necessary for them, it’s not good for them. There is no proof that it is helping the system, so you are actually pushing something on people that is in no shape or form proven to work or have any good effect in any shape or form and they don’t want to do it to their bodies. **Personally you’re putting something in somebody’s body that they don’t believe in, I don’t think you can actually bribe that**, I really don’t think you should do that. | If anybody would be open to having it, or opportunities were there to actually get it, it would it make it a lot easier, yes possibly. And that would make it quite user friendly but the people that wanted a flu vaccination had already had it before Flu Care started. |
| **M013_CH-H** | We had some posters, yes, we had some brochures, yes, we had that kind of stuff.  I think my staff were reading about it, I think I haven’t read it yet, to be honest. | **No. clicks:0**  **Average view duration: 00:00** | **No. clinics delivered: 2**  **No. staff vaccinated: 0** | Yes, basically, because I never had it before and **I really don’t want to have it**. **My residents have it and they get poorly afterwards anyway** so for me, I know it might be silly, but I’m just thinking what is the point if I have to get poorly anyway. So, yes, probably this is something that I would just through flu or cold and then, yes, just take some tablets and obviously a couple of days off and I will be fine again. **I don’t really want to get anything to my body, you know, I know it’s something different like a COVID injection but still I’d rather just get it and get through it**. Yes, to be honest, I think it’s good for the adults but for the older people like my residents, I’m just taking another example, because they need it more than me, I’m nearly 50 but my immune system is quite strong so it’s not very often I’m getting the flu jab then the flu. **I don’t think I need it, I think they need it more than me, maybe when I get** older I will start thinking about it more. But at the moment I feel fine, I feel healthy, I’m eating healthy. Yes, I try to do anything to avoid getting any cold and flu. My staff usually, well, they are young people, they don’t really want to have it yet because they’re afraid and they’re thinking they don’t need it. Obviously, it’s good to have it because we’re protecting not just ourselves it’s for residents as well. **So we want to make sure the residents are fine we provide the flu injections for** them. But for us because like I said we are staff and quite healthy so, you know, you can’t force anyone and at the moment when I talk to them most of them say, no, we don’t want anything at the moment, maybe when we get older but not at the moment I don’t want to have it." |  |
