# Supplementary material for: Process evaluation of the flucare cluster randomised controlled trial: assessing the implementation of a behaviour change intervention to increase influenza vaccination uptake among care home staff in England
Source: BMC Health Serv Res. 2025 Aug 21;25:1118. doi: 10.1186/s12913-025-13298-0 (PMC12369172; doi:10.1186/s12913-025-13298-0)
Supplement: Supplementary file 7 — Supplementary Material 7. [file 12913_2025_13298_MOESM7_ESM.docx]

**Staff engagement**

Engagement of staff with FluCare components (i.e., information materials (Posters, Leaflets and videos) and clinics has been mapped into four categories. The tables below provide illustrative quotes of the categories.

**Category 1: Engagers with all components of the intervention (i.e., information materials (either poster OR leaflet or video) AND clinic)**

| **Staff ID_Care Home ID** | **Poster and leaflet** | **Video** | **Clinics** | **Staff attitude regarding flu vaccinations** |
| --- | --- | --- | --- | --- |
| **S001_CH-J** | Yes**, I saw one leaflet**, I came to … I took one FluCare…, that time I received one. When I accepted yes, **I read that one**. | **Yes, I talked with my colleague because of this spreading to other residents, that is why the FluCare vaccination is very good**, yes | Yes, very easy to get it [the through FluCare clinics), yes. It is very easy I think, yes.  **Interviewer:** Do you normally get the flu vaccine each year?  No, first time I took this flu vaccination. My colleagues all are taking it from this care home. | **Interviewer:** **Do you normally get the flu vaccine each year**?  001: **No, first time I took this flu** |
| **S002_CH-L** | Yes, I did (see the posters) It made me realise that obviously it was available and I should probably do it… but yes, other than that I didn’t probably take too much looking at them, no.  **If we could maybe have leaflets available like during the clinic… But other than I think, yes, it did the job, it got me to have the flu vaccine, which I probably wouldn’t have done**. So, in that respect it did it, because it was there. So, yes, but the knowledge side of it I would have probably liked a bit more. But, as I say, it might just be that I didn’t look at it. **If there was leaflets and stuff around I might have just not seen it**. | I think so, I can’t remember now [seeing the video]. It would have been a while ago, wouldn’t it?. I think – yes, **I think I got sent something that I watched, or I did**. | **Interviewer:** So, you’ve told me that you have had the vaccine and you had it as part of the clinic within the care home?  **002: Yes.** **Interviewer:** If they hadn’t come into the clinic, do you think you would have got the flu vaccine just, say, with the materials around?  **002: No, probably not**. It’s, kind of, always something that’s in the back of my head, I probably need to do that, but then just life happens, and I just forget. So, if it’s not – but because they were there and I got the notice, they were like, “They’re coming in this day,” and I was like, “Great, I’m working, I’m here, I’ll go and get it.” And then I prioritised it on that day, but**, if it was up to me and I had to go and get it at the doctor’s, I highly doubt I probably would have managed to have gone and done it.** | I think I’ve only actually had it [flu vaccine]when I was pregnant, yes. If it was just for me I think I probably personally don’t need it. I’m quite young, I’m quite healthy, like my husband doesn’t get it because he’s not entitled to it. So, **I only would have it because of where I work, because I can**. I had it when I was pregnant, I’ve been pregnant twice now and I had it both times…, I think **previously to that I hadn’t necessarily, just because I’ve worked in a care home, and then this year I got it because they came in, so it was that convenient that they came in**. I probably see it quite the same, really, they’re (covid and flu vaccine) needed for like the vulnerable, but not necessarily for everybody. But I still probably don’t fully understand how me working in a care home…I don’t understand the connection. If it’s to help me, if I was to get sick, **I don’t understand why I should have it because I work in a care home**. |
| **S003_CH-A** | Well, to be **honest I already knew about it [flu vaccinations] before I saw the poster, so it just reminded me that it was there in the background** as something I was going to take part in…I can’t actually remember what was on it now, to be honest. | Interviewer: So, did you see the video about the flu vaccinations?  003: No. | To be honest up until this year I’ve never had one | A very personal choice thing, isn’t it? To be honest **up until this year I’ve never had one, but obviously once I’m working in a care home and I don’t want to take anything in there that could harm the residents, then obviously I’m going to take any precaution I can**. So, I’ve had obviously all the COVID vaccines and then the flu vaccine when that came out this year. I never felt that it was a threat to me, I don’t think I’ve ever actually had proper flu. Because people say it absolutely floors you and I’ve had bad colds, but I don’t think I’ve ever had flu. So, it wasn’t something I particularly worried about. |

**Category 2: Engagers with FluCare clinics**

| **Staff ID_Care Home ID** | **Poster and leaflet** | **Video** | **Clinics** | **Staff attitude regarding flu vaccinations** |
| --- | --- | --- | --- | --- |
| **S008 -CH-I** | **It made it more aware that the flu vaccine was available**…So, yes, I think, you know, the posters were quite open about when it was going to happen and where you could phone for further information. | No, I haven’t seen the video. | I would have had the flu vaccine anyway, but **it was just more convenient for me** to have it within my workplace | Well, **I’m for having the flu vaccine**. I think the majority of people should have it **because it does protect yourself** and flu is very unpleasant. |
| **S004_CH-J** | **I actually agreed with every single one of them. Yes, it is to protect yourself, but it’s to protect the vulnerable more**, you know? It’s like with all the COVID vaccines, yes, we all got fed up with them but it’s not about you, it’s about protecting others, as well. |  | **I have always been for the flu vaccines**. **I’ve been caring for 30-odd years now and I’ve been begging and pleading for flu jabs for care staff for years** | **I have always been for the flu vaccines. Because I’ve had family members that have been vulnerable** and you don’t want to go near them when you’ve got a cold, so no, I need to be near my family members. **Even my old ladies, you know…Because I’ve been caring for 30-odd years now and I’ve been begging and pleading for flu jabs for care staff for years**. The doctors have turned round and said, “Hey, you don’t need them,” and it’s only when you have health conditions that you’re invited to have one. But, you know, it’s incredible how people can’t see the fact that if we’ve got a cold how it impacts the people that we care for. |

**Category 3: Engage with none of the components of the FluCare intervention**

| **Staff ID_Care Home ID** | **Poster and leaflet** | **Video** | **Clinics** | **Staff attitude regarding flu vaccinations** |
| --- | --- | --- | --- | --- |
| **S011_CH-H** | Actually, **I haven’t seen about that because like I said I will not be having the flu jab anymore because it’s better for me to be poorly for a few days not for a long time**. |  |  | I honestly**, I had a very bad experience with the flu jab because a few years ago I had the flu jab** and after the flu jab I was poorly for two weeks, very poorly, I had antibiotics. Since that I decided that I’m not going to have the flu jab anymore because it’s made me more damaged. Because sometimes when you’re going to catch it you catch it if not you’re not. After that flu jab I was very, very poorly, I couldn’t move from the bed, I was on the sick, so since that I decided I’m not going to have it anymore. |
| **S012_CH-H** | **I didn’t read it, if I’m honest**, no…. Yes, I’ve just been too busy, to be fair. |  |  | I think the **flu vaccines are good for people that need it the problem is is they get poorly after** the fact which obviously they need a bit more care and things like that. A bit of extra TLC but **obviously I know it’s like a live ingredient isn’t it… So it’s got part of the virus in it, a small amount of virus in it to build up our immunity, that’s what we we’ve always been taught.** |
| **S015_CH-E** | I probably have (seen the posters/ leaflets) but **I’m not going to lie I’ve not really took a great deal of notice**, to be honest |  |  | **Interviewer:** So what did you think about the flu vaccination clinic being in the care home, because we did have a clinic?  **015:** I think it should be down to individual choice, like I say the majority of them they want it. My own opinion on it is I don’t know, **I don’t know whether I think they’re effective or not**. I’m going to be honest, I don’t really have an opinion on it (flu vaccinations) at all. I mean, like my parents are still alive, they’re in their eighties, do you know, I encourage them to go and have the flu vaccine and things like that. **But I’ve never had it myself because I’ve never really had the flu**. |
| **S016_CH-D** | **No, [haven’t seen posters and leaflets]** the only information I got was from my manager asking us it wasn’t compulsory but if anyone would like to take part in this project. | Interviewer: Yes. Did you by any chance see the video about flu or flu vaccinations?  016: No, I didn’t. |  | **I’ve never had a flu vaccine in my life, I’ve been fortunate that I’ve never had flu**. So probably the only way I might change my mind is statistics [state that it] would reduce the risk of me getting flu. **I’m 59 years of age and all that time I’ve never had a flu vaccination.** I’ve never suffered with flu, that’s not to say that I don’t see the importance of the flu vaccination because **I know it is important for a lot of people from different walks of life and different health conditions**. |
| **S017_CH-A** | Interviewer: did see the posters and leaflets about the flu vaccination?  017: Yes.  Interviewer: Did they have any effect on you?  017: No.  Interviewer: How come?  017: **The easiest way to put it is I don’t believe in the flu vaccine**.  **I don’t know if there can be more information because I didn’t really read them but I could see they were quite informative** but, I mean, percentages and things like that, people in the care industry or this home literally just look at statistics more than anything else. **From the information that I looked into it’s only in a certain percentage that it’s effective and it all depends what flu virus is going around as to whether it’s effective or not**. | Interviewer: Yes. Did you by any chance see the video as well?  017: No. |  | **I’ve never had flu and I’ve been around people that have had flu** and I’ve never had the vaccine either. |
| **S014_CH-I** | 014: I think I’ve seen a poster on a board at work.  Interviewer: Did the poster or leaflets have any effect on you?  014: Not particularly, no. **I don’t think I paid that much attention to the poster itself really**. | Interviewer: How about the video, did you see the video?  014: No. | I think it’s good that they come in and I think that makes it easier for people, **I just didn’t happen to be there on the day that it happened this year**. | Well, I’ve had it before and I know that this is anecdotal and **a lot of people say this but I always when I’ve had it have felt poorly straight afterwards. And then this year I sort of thought, well I never even get sick anyway so I’m just not going to get it**. That’s basically how I’ve felt about it this year but it’s sort of year to year. |

**Category 4: Staff already pro-vaccine (and had the vaccine elsewhere) before FluCare**

| **Staff ID_Care Home ID** | **Poster and leaflet** | **Video** | **Clinics** | **Staff attitude regarding flu vaccinations** |
| --- | --- | --- | --- | --- |
| **S009_CH-I** | When there is a group of us in the staff room **you look at the leaflets and you start to have a discussion**…And there are still people now that are, oh, I am not going to have the injection, and then there are others saying, well I am. Because, at the end of the day, you know, especially since COVID has been around as well, to me **if they’re giving you something that you haven’t got to pay for me personally I’ll just take i**t. | No, I haven’t seen the video, no. | I just live at the back of … my chemist is just out there. **I could have had it at work but I was off work that day but I just walked through to my chemist, it’s just five minutes**. **I’ve had it for the last three years nothing has happened to me, I haven’t had any symptoms like it’s knocked me off my feet or I’ve had to go off sick or anything like that**. I think it’s just going round and being confident in yourself that you’ve had it and then not putting a negative edge on something. | All the residents were having it and I’m thinking, what about if I get a cold…So **working in a care setting you’re going to work and then you think to yourself, hold on a minute, I can fight off this runny nose and cough and things like that but what about if I’m passing it on to these old folk** that are in their nineties. |
| **S005_CH-L** | Well, to be honest **I’d already had my flu jab**, I was quite early, so I’d already had it. So, it didn’t really – **obviously it looked, you know, interesting, but I’d already had my flu ja**b. So, yes. | Interviewer: were you shown the FluCare video?  005: I don’t think so, no. Not that I can remember. No, I don’t think we were. | Interviewer: If you had known that they would, come to the care home, do you think you would have waited  005: **yes, I probably would have waited. Yes, it probably would have been easier to have it done at work**… it would have been easier probably. Because I think I was on shift that day, so yes. | **I think I feel as if it’s part of my responsibility to have the flu jab because, like I said before, I wouldn’t want to give it to anyone else and I wouldn’t want it myse**lf. |
| **S007_CH-C** | To be honest **I didn’t take much notice**. **I just thought it was mainly for the – aimed at the residents more so than myself.** | Interviewer: did you see a video at all?  007: **To be honest I can’t remember**. | Interviewer: Did you have your vaccine in the clinic that we’d set up to happen in the care home.  007: **I think I did, but I can’t honestly remember. Either here or the pharmacy**. | To be honest I didn’t think at my age – I didn’t think I needed it, because I thought, well, I don’t suffer with asthma, I don’t have COPD, I’m not that old yet, but obviously **because I’ve turned over 60-plus then I’m at that category that it can affect me. So, yes, it’s a good thing**. To provide people or get people to have the flu injection rather than catch the actual flu virus. I do understand that you can still catch flu, but not get as much side-effects that you would normally do if you get – but **I’ve only had this since I’ve been working in care. Because up until then – and age I think is what it’s come to with me, is the age side of it.** |
| **S006_CH-C** |  |  | No, **I’d already had it two years previous**, so I’d planned not to have another COVID because I’ve had enough of those. **I just thought I will make sure I have my flu vaccine and I should be fine**. So, that’s the route I took. | For me I have it now because I’m approaching 60… until the last couple of years I’ve never bothered. Because I didn’t really get ill like that, I’m quite healthy. But because I’m getting older and Mum’s more vulnerable I just thought it’s important, hence why I’ve had it. Not really work-related at all, selfish reasons [laughs]." |
